# The association between aggression and risk of Internet gaming disorder in Korean adolescents: the mediation effect of father-adolescent communication style

**DOI:** 10.4178/epih.e2018039

**Published:** 2018-08-08

**Authors:** Eunjin Kim, Hyeon Woo Yim, Hyunsuk Jeong, Sun-Jin Jo, Hae Kook Lee, Hye Jung Son, Hyun-ho Han

**Affiliations:** 1 Department of Preventive Medicine, College of Medicine, The Catholic University of Korea, Seoul, Korea; 2 Department of Psychiatry, College of Medicine, The Catholic University of Korea, Seoul, Korea

**Keywords:** Addictive behavior, Aggression, Communication, Mediation

## Abstract

**OBJECTIVES:**

Open and supportive communication between parents and children is known to reduce adolescents’ delinquent behavior. Recently, the risk of Internet gaming disorder (IGD) has been increasing in adolescents. The purpose of this study was to investigate the mediating effects of parent-child communication styles on the relationship between adolescent aggressiveness and risk of IGD.

**METHODS:**

Participants in this study were 402 first-year students from 4 middle schools in Seoul who enrolled in the Internet user Cohort for Unbiased Recognition of gaming disorder in Early adolescence (iCURE) and completed baseline assessment in 2016. The structural equation model was constructed based on an aggression questionnaire, the Internet game use-elicited symptom screen, a mother-child communication inventory, and a father-child communication inventory.

**RESULTS:**

Adolescents’ aggressiveness was found to be related to their risk of IGD. The father-child communication style mediated the relationship between aggression and risk of IGD. However, the mother-child communication style had no mediating effect.

**CONCLUSIONS:**

Our findings suggest that fathers should make an effort to improve open and positive communication skills with their children, because the father-child communication style plays an important role in the relationship between adolescent aggressiveness and risk of IGD.

## INTRODUCTION

During the stormy period of adolescence, teenagers undergo development to adulthood and begin to exhibit problematic behaviors or negative emotions. In adolescents, prefrontal development is still incomplete, and the amygdala, which controls fear and anger, plays a larger role in behavior; therefore, adolescents are more prone to exhibiting impulsive behaviors. In addition, adolescents’ bodies release testosterone, the male hormone, greater than are released during the juvenile period, and this hormone stimulates their aggression and anger to make them more aggressive [1]. Aggression is an intended behavior that harms or hurts others and includes the angry emotions that lead to aggressive behaviors [2].

Aggression increases during adolescence and then consistently decreases as one enters adulthood [3]. Aggression during adolescence has been reported to be associated with antisocial behaviors such as delinquency, addiction, and violence as well as with crime and school maladjustment. Families affect adolescents’ socialization and can thus play an important role in adolescents’ antisocial and criminal behaviors. Parents play the most important role in adolescents’ process of socialization [4]. Communication between adolescents and parents is known to be positively associated with adolescents’ achievement [5], self-esteem [6], and psychological health [7], and negatively associated with loneliness, depression [6], drug abuse, and delinquent behaviors [8]. Open and free parent-child communication helps prevent delinquent behaviors, whereas closed and repressive communication negatively affects the child and leads to delinquency [9].

Owing to the rapid development of information and communication technology in recent years, Internet games have become popular through high-speed Internet. Internet games have become a part of leisure activities enjoyed by children and adults, but addiction to Internet games can lead to a variety of social, physical, and psychological problems [10]. One study has shown that Internet gaming disorder (IGD) causes the same brain changes as those caused by alcohol and drug addiction [11]. The frontal lobe plays the most important role in self-recognition, behavior planning, information integration, and storing of emotions, impulses, and desire. It slowly develops between 12 and 20 years of age at the latest; thus, such brain changes affect adolescents more than they affect adults [12].

Middle school students are the most frequent users of Internet games. A survey of overdependence on the Internet has shown that adolescents occupy the highest proportion (30.6%) of the high-risk overdependence group among all age groups [13]. The risk factors of IGD that have been identified to date include stress, depression, anxiety, impulsiveness, aggression, and financial status. Of these, aggression is known as one of the causes of IGD [14-16].

Thus, this study was conducted to investigate whether or not open and free parent-child communication has a mediating effect on the risk of IGD among adolescents, since parents play a mediating role in adolescents’ delinquent and violent behaviors.

## MATERIALS AND METHODS

### Subjects

The Internet user Cohort for Unbiased Recognition of gaming disorder in Early Adolescence (iCURE) was established to investigate the characteristic of IGD and observe the natural progress of IGD among third and fourth graders in primary school and first-year students in middle school residing in Seoul and Gyeonggi Province [17]. This study included 440 first graders in 4 middle schools located in Seoul that were surveyed between April 6 and May 26, 2016, during the iCURE cohort research. All 4 schools were coeducational.

A structural equation model was analyzed to answer the research question. For the sample size of the structural equation model, 10-20 subjects are needed for the estimation of each parameter [18]. In this study, 20 parameters were to be estimated using a model constructed with 4 measurements, and thus a sample size of over 400 was planned.

Baseline data about aggression, risk of IGD, and mother- and 2father-child communication styles were extracted from the iCURE cohort data and analyzed. Thirty-eight cases with missing data due to the absence of a mother or father were excluded, and the data of 402 subjects were finally used.

### Ethics statements

This study extracted and analyzed data from the iCURE cohort study and was approved by the institutional review board of the Catholic University of Korea (no. MC17EESI0073).

### Methods of investigation and tools

A web-based self-reported survey was conducted during school hours. The following tools were used.

#### Aggression

The Buss-Perry Aggression Questionnaire (AQ) was used to investigate aggression [19]. This scale consists of 27 questions and 4 dimensions of aggression (physical aggression, verbal aggression, anger, and hostility). It is a 5-point Likert scale with scores ranging from 1 (never) to 5 points (very true). Total scores ranged from 27 to 135 points, with higher scores indicating higher levels of aggression. In this study, the internal consistency of this tool (Cronbach‘s α) was 0.88. The Korean version of the AQ was used in this study [20].

#### Risk of Internet gaming disorder

The Internet Game Use-Elicited Symptom Screen (IGUESS) was used to measure the risk of IGD. The IGUESS is a self-reported scale used to screen symptoms of IGD based on the fifth edition of the Diagnostic and Statistical Manual of Mental Disorders (DSM-5) suggested IGD diagnostic criteria. It consists of nine questions rated on a 4-point Likert scale, with total scores ranging from 0 to 27 points. Higher scores indicate a higher risk of IGD. In this study, the internal consistency (Cronbach‘s α) of this tool was 0.86 [21].

#### Style of parent-child communication

The Parent-Adolescent Communication Inventory (PACI) developed by Barnes & Olson [22] was used to assess the style of parent-child communication. The tool consists of 20 questions with two types of communication: open communication and problematic communication. The questions are rated on a 5-point Likert scale with scores ranging from 1 (never) to 5 points (very true) for each question. Total scores range from 20 to 100 points, with higher scores indicating more open and positive communication and lower scores indicating more dysfunctional and negative communication. The internal consistency of this tool (Cronbach‘s α) was 0.91 in this study. The Korean version of the tool was used in this study [23].

### Statistical analysis

SPSS version 24 (IBM Corp., Armonk, NY, USA) was used for descriptive statistics and correlational analysis. AMOS version 23 (IBM Corp., Armonk, NY, USA) was used to analyze the measurement model and mediating effects using structural equation modeling. The research model was constructed based on the assumption that the style of father- or mother-child communication is a major mediating variable in the relationship between aggression and risk of IGD. IGUESS scores, which were one of the major variables, were found not to have a normal distribution in the normality test and were converted to log values. A correlational analysis was performed to analyze the correlations between aggression, risk of IGD, and style of mother- or father-child communication included in the research model and to check for multicollinearity. A confirmatory factor analysis, which is used to confirm inherent factor dimensions and hypotheses based on the researcher’s knowledge and to test the validity of measurement scales for a certain concept, was performed to evaluate the validity of each measurement parameter.

The construct validity of the major variables was tested in terms of convergent validity and discriminant validity. Convergent validity is the degree to which two or more measurement parameters correlate with one another with respect to a construct. A standardized factor loading of 0.5 is essential and one of 0.7 or higher is advisable. Average variance extracted (AVE) is the ratio of the sum of the squares of the factor loadings and that sum plus the sum of the factor error variances. In general, a model is deemed to have convergent validity when its AVE is 0.5 or over. This means that half of all variance must be explained by the construct in order to accept the parameters.

The validity of a structural equation model is determined by assessing the consistency between the research model and the actual covariance data and how fit the covariance structural model is for the assumption. In this study, the structural equation model was deemed fit under the conditions of a normed χ^2^, which is an absolute fit index that represents how well a research model reflects the enrolled data, of 3 or less; a goodness of fit index (GFI) of 0.90 or over; a root mean square error of approximation (RMSEA) of 0.05 or less; a comparative fit index (CFI), which determines how fit a model is in comparison with a null model in which no relationships are set between all variables of a model, of 0.9 or over; and a Tucker-Lewis index (TLI) of 0.9 or over.

To test the statistical significance of indirect effects, a bootstrap method was used in which 20,000 data samples randomly sampled from the original data were used for parameter estimation and 95% confidence intervals were calculated.

## RESULTS

### General characteristics

First-year 4 coeducational middle school students were included. Of the students, 223 (55.5%) were male. The mean aggression score was 57.4±15.0 points, the mean PACI score for father-child communication was 69.8±15.1 points, and that for mother-child communication was 63.0±13.2 points. On IGD risk scores, 77.8% had scores of 0-5 points, 13.2% scores of 6-9 points, and 9.0% scores of 10 points or over, which is the cut-off point of the IGD (Table 1).

### Correlational analysis

**Figure 1.**
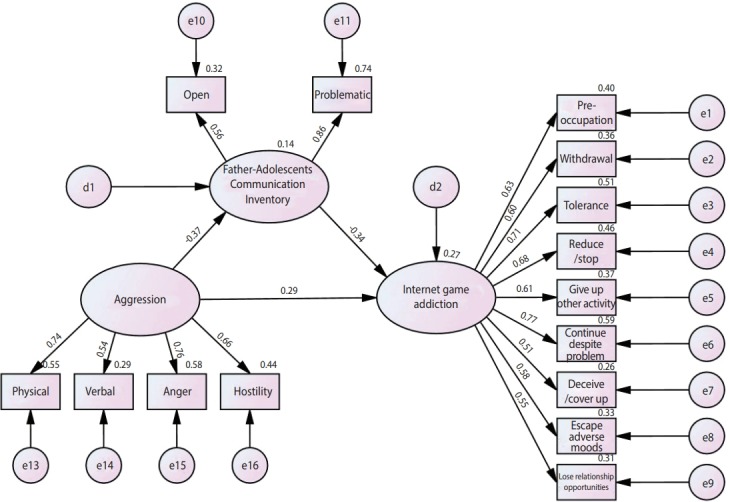
The mediating effects of father-adolescents communication style between aggression and risk of Internet game disorder. e, measurement error; d, structural error.

A correlation analysis of the major measurement parameters included in the research models (aggression, risk of IGD, and styles of father- and mother-child communication) showed a significantly positive correlation between aggression and the risk of IGD (r=0.32, p<0.001). Aggression was significantly negatively correlated with styles of father-child communication (r=-0.22, p<0.001), and styles of father-child communication were significantly negatively correlated with the risk of IGD (r=-0.38, p<0.001). Styles of mother-child communication did not significantly correlate with aggression (r=0.04, p=0.39), styles of father-child communication (r=0.03, p=0.61), or the risk of IGD (r=-0.05, p=0.30) (Table 2). Thus, a model was constructed with styles of father-child communication as a mediating factor in the relationship between aggression and the risk of IGD (Figure 1).

### Evaluation of measurement models

#### Confirmatory factor analysis

Confirmatory factor analysis yielded a construct reliability, which measures convergent validity, of 0.94 for IGD, 0.71 for styles of father-child communication, and 0.87 for aggression; these values satisfied the acceptable standard value of 0.7 or above. The mean AVE was 0.65 for IGD, 0.57 for styles of father-child communication, and 0.63 for aggression; these values satisfied the acceptable standard value of 0.5 or above, meaning that the model had convergent validity (Figure 1).

Regarding the discriminant validity of the major measurement parameters, the parameters had a mean AVE value greater than the square of their correlation coefficient below the diagonal on Table 3 and thus had discriminant validity (Table 3).

#### Structural equation model goodness of fit test

Based on the results of a goodness of fit test of the model with styles of father-child communication as a mediating factor in the relationship between aggression and IGD, the mediating effect and the model were deemed fit (χ^2^=201.52, df=87.00, χ^2^/df =2.32, GFI=0.94, RMSEA=0.06, CFI=0.94, TLI=0.92) (Table 4).

#### Direct, indirect and total effects

Analysis of the structural equation model showed that the direct effect of aggression on IGD was 0.29 and was statistically significant (p<0.001). The indirect effect of styles of father-child communication in the relationship between aggression and the risk of IGD was 0.13. The significance of indirect effects was tested using the bootstrap method, and the path through which styles of father-child mediated the relationship between aggression and the risk of IGD was statistically significant (p<0.001). Thus, it was found that styles of father-child communication partially mediated the effect of aggression on the risk of IGD, and their total effect was found to be 0.42 (p<0.001) (Table 5).

## DISCUSSION

The mean aggression score measured by the AQ developed by Buss & Perry [19] was 57.34±15.00, which is somewhat lower than scores observed in previous studies [24]. The AQ defines aggression as the tendency to harm or hurt others, and aggression includes physical and verbal aggression toward others and dangerous ways of thinking and emotions. This reflects the impulsiveness or aggressive desire that adolescents experience during the process of adjusting to hormonal and environmental changes in the transitional period. The subjects in this study were first-year students in middle school. Assuming that they had not reached the peak of puberty, it is reasonable to argue that they had lower aggression scores than subjects in other studies that included the entire population of middle school students or included high school students. In a cohort study that included 5,151 adolescents aged 11-18, physical aggression reached a peak before and after the age of 15 years, and social aggression reached a peak before and after the age of 14. Adolescents tended to show low levels of aggression in early adolescence that gradually increased and then decreased with age [25].

Biological factors have been reported to affect aggression more significantly than environmental factors [26]. Brain imaging results have shown that aggression is associated with a reduced volume of gray matter in the medial prefrontal cortex and lateral frontal cortex. Reduced gray matter in the prefrontal region is associated with aggressive behaviors regardless of diagnosis of psychological disorder, and this suggests that aggression is affected by biological factors since many genetic factors come into play in the gray matter of the frontal cortex [27]. A long-term twin follow-up study showed that genetic factors play a major role in social aggression [28,29].

The DSM-5 diagnoses IGD when at least five of nine symptoms are observed. The score range for the IGUESS is 0-27 points with a cut-off point for the risk of IGD of 10 points or greater. In the IGUESS, two points are given for symptoms that occur “frequently.” Scores of 10 points or greater thus correspond to displaying five of the nine symptoms in the DSM-5 diagnostic criteria for IGD [21]. In this study, 9.0% of all subjects had the cut-off score of 10 points or greater.

A significant association was found between aggression during adolescence and the risk of IGD (r=0.32, p<0.001). This finding was consistent with a previous report that aggression affects the risk of IGD [30-32]. Thus, higher levels of aggression during adolescence indicate a higher likelihood of being included in the high-risk group for IGD.

This study investigated whether or not styles of parent-child communication can decrease the risk of IGD in the relationship between aggression and the risk of IGD and found that styles of mother-child communication had no mediating effects on the relationship between aggression and the risk of IGD, whereas styles of father-child communication did.

Analysis of the structural equation model showed that the total effect of aggression on the risk of IGD was 0.42, of which 0.29 was the direct effect and 0.13 was an indirect effect mediated by father-child communication. Standardized path coefficients of 0.1-0.3 indicate small effects, those of 0.3-0.5 medium effects, and those of 0.5 or greater large effects. Thus, father-child communication can be considered as having a medium to large effect on the association between adolescents’ aggression and the risk of IGD. Prospective randomized clinical trials are needed to investigate whether encouraging open parent-child communication contributes to reducing problematic behaviors including IGD.

Communication is a complex and dynamic interactive process. According to a theory comparing parental roles, in a traditional family the father plays an “instrumental role” as the family’s representative, whereas the mother plays an “expressive role” of satisfying emotional needs. During the process of raising children, the father plays a controlling role in his children’s behaviors, and the mother plays a nurturing role by providing affection through words, behavior, and physical contact. When playing with his/her father, a child can learn that physical violence such as kicking and biting is not socially acceptable as well as learn the proper balance between timidity and aggression [33]. Considering that parents have different impacts on their children depending on their genders, the risk of IGD may be more associated with fathers. In a British cohort follow-up study, fathers’ involvement and attitude in early adolescence had an important impact on children’s psychological health [34]. Although it is generally known that mothers have a significant impact on their children, the reason that fathers had a greater impact on the association between aggression and the risk of IGD may be that the study participants were in the first year of middle school. Further research is necessary to determine whether the results of this study are phenomena that occur during a specific period or throughout adolescence.

The effect of environmental factors on aggression varies depending on gender, with females affected relatively less by the environment [29]. To investigate the differences in the mediating effect of father-child communication on the association between aggression and the risk of IGD, a multi group structural equation model stratified by gender was analyzed. No sex differences were found, and styles of father-child communication were identified as a mediating factor for both males and females (results not provided).

This study has several limitations. First, since it is a cross-sectional study, it cannot analyze the chronological relationship between adolescents’ aggression, styles of parent-child communication, and IGD. Longitudinal studies must be conducted to understand the causal relationships between these variables.

Second, there is a possibility of misclassification since the diagnostic criteria for IGD have not been clearly established. However, the IGUESS, which classifies risk of IGD under conditions for further study in DSM-5, was used to increase the reliability and validity of such tests. Since the IGUESS is based on self-reported responses, the possibility of false positives and false negatives cannot be eliminated.

Third, the results of this study are based on subjects from four middle schools in Seoul who were selected by convenience sampling, and since the subjects were in their first year of middle school, the results cannot be generalized to the entire population of middle school students.

Previous studies evaluated the combined effects of parent-child communication styles in mediating effect on relationship between aggression and gaming addiction. However, we distinguished between mothers and fathers in parent-child communication to analyze the mediation effect using a structural equation model.

In this study, aggression during adolescence was associated with the risk of IGD. The mediating effect of father-adolescent communication observed in this study suggests that efforts for open and positive communication between fathers and adolescents are necessary.

## Figures and Tables

**Table 1.** Baseline characteristics of 402 adolescents

Characteristics	Mean±SD	n (%)
Age	13.0±0.4	
Sex (male)		223 (55.5)
School type (coeducation)		4 (100.0)
IGUESS		
0-5		313 (77.8)
6-9		53 (13.2)
≥10		36 (9.0)
AQ	57.4±15.0	
PACI (father)	69.8±15.1	
PACI (mother)	63.0±13.2	

SD, standard deviation; IGUESS, Internet Game Use-Elicited Symptom Screen; AQ, Aggression Questionnaire; PACI, Parent-Adolescent Communication Inventory.

**Table 2.** Correlation matrix of the independent, mediation, and dependent variables^1^

	1	2	3	4	Mean	Standard deviation
1. Aggression	1.00				57.34	15.00
2. Father-adolescent communication style	-0.22^***^	1.00			69.80	15.06
3. Mother-adolescent communication style	0.04	0.03	1.00		63.01	13.15
4. Risk of Internet game disorder	0.32^***^	-0.38^***^	-0.05	1.00	3.61	4.00

1 Aggression was evaluated by Aggression Questionnaire; Father- and mother-adolescent communication styles were assessed by Parent-Adolescent Communication Inventory; Risk of Internet game disorder was measured by Internet Game Use-Elicited Symptom Screen.

*** p<0.001.

**Table 3.** Variance shared between constructs

	Aggression	Father-adolescent communication style	Risk of Internet game disorder
Aggression	0.63		
Father-adolescent communication style	0.05	0.57	
Risk of Internet game disorder	0.10	0.14	0.65

Aggression was evaluated by Aggression Questionnaire; Father- and mother-adolescent communication styles were assessed by Parent- Adolescent Communication Inventory; Risk of Internet game disorder was measured by Internet Game Use-Elicited Symptom Screen.

**Table 4.** The model fit of the independent, mediation, and dependent variables

Index	χ^2^	*df*	χ^2^/*df*	GFI	RMSEA	CFI	TLI
The mediation model	201.52	87.00	2.32	0.94	0.06	0.94	0.92

GFI, goodness of fit index; RMSEA, root mean square error of approximation; CFI, comparative fit index; TLI: Tucker-Lewis index.

**Table 5.** Direct and indirect effects on risk of IGD

Path	Effect^***^		
	Total	Direct	Indirect
Aggression → risk of IGD	0.42	0.29	0.13

GD, Internet game disorder.

*** p <0.001.

## SUPPLEMENTARY MATERIALS

Supplementary Material: Korean version is available at http://www.e-epih.org/.
