# Supplementary material for: The association between aggression and risk of Internet gaming disorder in Korean adolescents: the mediation effect of father-adolescent communication style
Source: Epidemiol Health. 2018 Aug 8;40:e2018039. doi: 10.4178/epih.e2018039 (PMC6232655; doi:10.4178/epih.e2018039)
Supplement: Supplementary file 1 [file epih-40-e2018039-supplementary.pdf]

Volume: 40, Article ID: e2018039
https://doi.org/10.4178/epih.e2018039

The association between aggression and risk of Internet gaming disorder in Korean adolescents: the mediation effect of father-adolescent communication style

Eunjin Kim[1], Hyeon Woo Yim[1], Hyunsuk Jeong[1], Sun-Jin Jo[1], Hae Kook Lee[2], Hye Jung Son[1], Hyun-Ho Han[1]

[1]Department of Preventive Medicine, College of Medicine, The Catholic University of Korea, Seoul, Korea; [2]Department of Psychiatry, College of Medicine, The Catholic University of Korea, Seoul, Korea

Corresponding author

Hyeon Woo Yim

Department of Preventive Medicine, College of Medicine, The Catholic University of Korea, 222 Banpo-daero, Seocho-gu, Seoul 06591, Korea

E-mail: y1693@catholic.ac.kr

Volume: 40, Article ID: e2018039
https://doi.org/10.4178/epih.e2018039

ABSTRACT

OBJECTIVES: Open and supportive communication between parents and children is known to reduce adolescents' delinquent behavior. Recently, the risk of Internet gaming disorder (IGD) has been increasing in adolescents. The purpose of this study was to investigate the mediating effects of parent-child communication styles on the relationship between adolescent aggressiveness and risk of IGD.

METHODS: Participants in this study were 402 first-year students from four junior high schools in Seoul who enrolled in the Internet user Cohort for Unbiased Recognition of gaming disorder in Early adolescence (iCURE) and completed baseline assessment in 2016. The structural equation model was constructed based on an aggression questionnaire, the Internet game use-elicited symptom screen, a mother-child communication inventory, and a father-child communication inventory.

RESULTS: Adolescents' aggressiveness was found to be related to their risk of IGD. The father-child communication style mediated the relationship between aggression and risk of IGD. However, the mother-child communication style had no mediating effect.

CONCLUSIONS: Our findings suggest that fathers should make an effort to improve open and positive communication skills with their children, because the father-child communication style plays an important role in the relationship between adolescent aggressiveness and risk of IGD.

KEY WORDS: Internet game disorder, Aggression, Communication, Mediation

INTRODUCTION

흔히 '질풍노도의 시기'로 불리는 청소년기에는 발달 과업을 이루는 시기인 동시에 문제행동이나 부정적 감정을 표출 할 수 있는 시기이다. 청소년기는 전전두엽의 발달이 미숙하고, 공포와 분노를 조절하는 편도체가 더 큰 역할을 하여 충동적인 행동 반응을 보이기 쉽다. 또한, 남성 호르몬인 테스토스테론 분비가 소아기보다 증가되며 청소년의 공격성과 분노를 자극하여 유달리 쉽게 공격적이 된다 [1]. 공격성은 다른 사람에게 해를 입히거나 상처를 주는 의도적인 행동이며[2], 이러한 공격적 행동을 야기하는 분노 감정을 포함하여 정의할 수 있다 [2].

공격성향은 청소년기에 증가하였다가 이후 성인기에 들어감에 따라 지속적으로 감소하는 양상을 보인다 [3]. 청소년기의 공격성은 비행, 중독, 폭력 등의 반사회적 행위 및 범죄와 학교부적응과의 연관이 보고된 바 있다. 가족은 청소년의 사회화에 영향을 미치므로 이러한 반사회적 행위 및 범죄 행동에 가족이 중요한 역할을 할 수 있다. 부모는 사회화 과정에서 가장 중요한 역할을 한다 [4]. 청소년과 부모와의 의사소통은 청소년의 성취 [5], 자아존중감 [6], 정신건강 Collins [7]과 정

적인 관계가 있고, 외로움, 우울증 [6], 약물남용 및 비행행동 [8]과 부적 관계가 있다고 알려져 있다. 부모-자녀 간 의사소통이 개방적이고 자유로운 의사소통을 하는 경우 비행 행동을 예방하나 부모-자녀 간 폐쇄적이고 억압적인 의사소통을 하는 경우 부정적으로 작용하여 비행으로 이어질 수 있다 [9].

최근 인터넷 및 정보 통신 기술(Information & Communication Technology, ICT)의 비약적인 발전을 기반으로 초고속 인터넷을 통한 인터넷 게임이 대중화되고 있다. 인터넷 게임은 유아부터 성인까지 즐기는 여가생활의 일부로 자리잡았으나 지나치게 이용하여 중독이 되는 경우 사회적, 신체적 및 정신적 건강 관련 여러 가지 문제가 발생하고 있다 [10]. 인터넷게임장애(Internet gaming disorder, IGD)가 알코올 중독과 마약 중독과 동일한 뇌 변화를 일으킨다는 연구결과가 있었다 [11]. 전두엽은 자기를 인식하고, 행동을 계획하고, 각종 정보를 통합하며, 감정, 충동, 욕구를 저장하는 뇌에서 가장 중요한 역할을 하는데, 12세부터 늦으면 20세 이후까지 가장 천천히 성숙하기 때문에 이러한 뇌 변화는 성인보다 청소년에게 더욱 큰 영향을 미친다 [12].

현재 중학생이 인터넷 게임을 가장 많이 이용하고 있으며, 인터넷 과의존 실태조사 결과, 문제가 될 가능성이 높은 과의존위험군은 청소년이 30.6%로 다른 연령대에 비해 가장 높았다 [13]. 현재까지 밝혀진 청소년 인터넷게임장애 위험성과 관련된 위험요인으로 스트레스, 우울, 불안, 충동성, 공격성, 경제수준 등이 있으며, 그 중 공격성이 하나의 원인으로 알려져 있다 [14-16].

따라서, 본 연구에서는 청소년에서 부모의 역할이 비행 및 폭력 행동을 매개하듯이 청소년에서 부모와 개방적이고 자유로운 의사소통이 인터넷게임장애 위험에 매개효과가 있는지 확인해보고자 한다.

MATERIALS AND METHODS

2.1. 대상자

서울과 경기도에 거주하는 초등학교 3,4학년생과 중학교 1학년생을 대상으로 인터넷게임장애의 특성을 파악하고 인터넷게임장애의 자연사를 관찰하기 위한 인터넷 사용자 코호트 연구(the Internet user Cohort for Unbiased Recognition of gaming disorder in Early Adolescence, iCURE)가 수행되고 있다 [17]. 본 연구는 iCURE 코호트 연구 등록자료 중에서 2016년 4월 6일부터 5월 26일까지 조사를 수행한 서울에 소재한 4개 중학교 1학년 440명을 대상으로 하였으며 4개학교 모두 남녀공학이었다.

연구질문에 대한 분석은 구조방정식 모형분석을 실시하였다. 구조방정식 모형분석을 위한 표본크기는 추정하고자 하는 모수당 10-20명의 대상자가 필요하다 [18]. 본 연구에서 4개 측정값으로 모형을 구성할 때 20개의 모수를 추정할 계획이므로 400명 이상의 표본 수를 계획하였다.

iCURE 코호트 기저시점 자료 중에 공격성, 인터넷게임장애 위험성, 어머니와 자녀 간의 의사소통

방식 및 아버지와 자녀 간의 의사소통 방식 자료를 추출하여 분석하였고, 어머니가 없거나 아버지가 없어서 자료 결측치가 있었던 38건을 제외하고, 총 402명의 자료를 최종적으로 사용하였다. iCURE 연구에서 자료를 추출하여 분석하는 본 연구는 가톨릭대학교 임상연구윤리위원회로부터 연구를 승인 받았다(승인번호 MC17EESI0073).

2.2. 조사방법 및 도구

조사방식은 웹 기반 자가보고로 학교 수업시간을 이용하여 조사되었으며 각 조사도구는 다음과 같았다.

2.2.1. 공격성

공격성은 Buss와 Perry가 개발한 (Aggression questionnaire. AQ)를 이용하였다 [19]. 이 척도는 공격성에 대한 4개의 하위요인(신체적 공격성, 언어적 공격성, 분노감, 적대감) 총 27문항으로 구성되어 있는 5점 likert scale로 1점(전혀 그렇지 않다)부터 5점(매우 그렇다)로 측정되었다. 점수의 범위는 27점부터 135점까지로 점수가 높을수록 공격특성이 높음을 나타낸다. 본 연구에서 내적일관성(Cronbach's α)은 .88이었고, 한국어로 표준화된 도구를 사용하였다 [20].

2.2.2. 인터넷게임장애 위험성

인터넷게임장애 위험성은 Internet Game Use-Elicited Symptom Screen (IGUESS)로 측정하였다. IGUESS는 DSM-5의 인터넷게임장애 (Internet Gaming Disorder) 진단기준에 근거한 인터넷게임장애 증상을 선별하기 위한 자가보고척도이다. 총 9문항의 Likert형 4점 척도로 구성되어 있으며, 점수의 범위는 0점부터 27점이다. 점수가 높을수록 인터넷게임장애 위험성이 높다고 볼 수 있다. 본 연구에서 내적일관성(Cronbach's α)은 .86였다 [21].

2.2.3. 부모와 자녀 간의 의사소통 방식

부모와 자녀 간 의사소통방식(Parent-Adolescent Communication Inventory, PACI)은 Barnes와 Olson(1982)가 개발한 도구를 사용하였다 [22]. 의사소통 방식은 개방적 의사소통과 문제형 의사소통 총 2개의 하위요인 20문항으로 구성되어 있으며, 5점 Likert 척도로 1점(전혀 그렇지 않다)부터 5점(매우 그렇다)로 평가한다. 점수의 범위는 20점부터 100점까지로 점수가 높을수록 의사소통이 개방적이고 긍정적이며, 점수가 낮을수록 의사소통이 역기능적이고 부정적임을 의미한다. 본 연구에서 내적일관성(Cronbach's α)은 .91이었고, 한국어로 번안된 도구를 사용하였다 [23].

2.3. 통계분석

Volume: 40, Article ID: e2018039
https://doi.org/10.4178/epih.e2018039

기술통계 및 상관분석은 SPSS(version 24)을 이용하였고, 구조방정식(Structural equation modeling, SEM)을 이용한 측정모델 및 매개효과 분석은 AMOS(version 23)를 이용하였다(IBM SPSS, Chicago, IL, USA). 연구모형은 공격성과 인터넷게임장애 위험성과의 관계에서 어머니 혹은 아버지와 자녀 간의 의사소통 방식이 주요한 매개변수로 가정하여 구조방정식 모형을 구성하였다.

주요 변수 중 하나인 IGUESS는 정규성 검정에서 정규분포를 하지 않은 것으로 확인되어 로그(Log) 변환하여 사용하였다.

본 연구모형에 포함된 공격성, 인터넷게임장애 위험성, 어머니 혹은 아버지와 자녀 간의 의사소통 방식 간의 상관관계 및 다중공선성을 확인하기 위해 상관분석을 실시하였다. 측정변수의 타당성 확인을 위해 확인적 요인분석(CFA)를 실시하였다. 확인적 요인분석은 연구자의 지식에 근거하여 내재된 요인차원 및 가설을 확인하는 수단으로 사용되며 특정개념에 대한 측정 척도들의 타당성을 평가하는데 유용한 분석기법이다.

주요변수의 구인타당도 검정은 수렴타당도(Convergent Validity)와 판별타당도(Discriminant Validity)로 검정하였다. 수렴타당도는 하나의 구성개념에 대한 두 개 이상의 측정도구가 상관관계를 갖는 정도에 관한 것으로 첫째, 표준화 요인부하량(Standardized Factor Loading)이 .5 이상이 되어야 하며, .7 이상이면 바람직하다. 둘째, 평균분산추출(Average Variance Extracted, AVE)은 표준화 요인부하량을 제곱한 값들의 평균으로, 표준화 요인부하량 값의 1/2은 그 구성개념에 의해 설명되고 나머지 1/2은 오차분산이 된다. 일반적으로 .5 이상이면 집중타당성을 갖는 것으로 받아들이는데, 이는 분산 중 1/2은 구성개념에 의해 설명될 수 있어야 그 항목들을 수용할 만하다는 의미가 된다.

구조방정식 모형의 적합성은 연구모형과 실제 공분산자료 사이의 일치성(Consistency)의 정도를 알아보는 것으로 공분산 구조모형이 가정에 얼마나 적합한가를 살펴보는 절차이다. 본 연구에서는 연구모형이 입력자료를 얼마나 잘 반영하는지를 나타내는 절대적합지수인 x^2통계량, Normed x^2 은 3 이하, 기초부합지수(Goodness of Fit Index, GFI)은 .90 이상, 근사원소평균자승 잔차(Root Mean Square Error of Approximation, RMSEA)는 .05 이하와 연구모형이 모든 변수가 관계가 전혀 설정되지 않은 영 모델(Null Model)에 비해 얼마나 적합도가 높은지 평가하는 증분적합지수인 비교적합지수(Comparative Fit Index, CFI) .9이상, Tucker-Lewis 지수(Tucker-Lewis Index, TLI)는 .9이상을 근거로 판단하였다.

간접효과가 통계적으로 유의한지 확인하기 위해 부트스트랩(Bootstrap) 방식을 사용하였다. 원 자료에서 무선 표집(Random Sampling)으로 생성된 20,000개의 자료 표본이 모수 추정에 사용되었으며, 95%의 신뢰구간을 산출하였다.

RESULTS

일반적특성

Volume: 40, Article ID: e2018039
https://doi.org/10.4178/epih.e2018039

4개 중학교 모두 남녀공학 1학년 학생이었고, 남학생은 55.5% 였다. 공격성은 평균 57.4(±15.0), 아버지와 자녀와의 의사소통 방식(PACI)은 평균 69.8(±15.1), 어머니와 자녀와의 의사소통 방식(PACI)은 평균 63.0(±13.2), 인터넷게임장애 위험성은 0-5점 사이 77.8%, 6-9점 사이 13.2% 절단점인 10점 이상은 9%로 나타났다(Table. 1).

Insert Table 1 here

상관 분석

본 연구모형에 포함된 주요측정변수 즉, 공격성, 인터넷게임장애 위험성, 어머니와 자녀와의 의사소통 방식, 아버지와 자녀와의 의사소통방식 간에 상관분석 결과 공격성과 인터넷게임장애 위험성은 유의한 정적 상관을 보였다($r=0.32$, $p<.001$). 공격성과 아버지와 자녀 간의 의사소통방식은 유의한 부적상관을 보였고($r=-0.22$, $p<.001$), 아버지와 자녀 간의 의사소통방식과 인터넷게임장애 위험성은 유의한 부적상관을 보였다($r=-0.38$, $p<.001$). 어머니와 자녀 간의 의사소통방식은 공격성($r=0.04$, $p=.39$), 아버지와 자녀 간의 의사소통방식($r=0.03$, $p=.61$), 인터넷게임장애 위험성 ($r=-0.05$, $p=.30$)과 상관관계가 유의하지 않았다 (Table 2). 그리하여 공격성과 인터넷게임장애 위험성과의 관련성에서 아버지와 자녀 간의 의사소통 방식만을 매개요인으로 고려하여 모델을 구성하였다(Figure 1).

Insert Table 2 here

측정 모델의 평가

확인적 요인분석(Confirmatory Factor Analysis, CFA)

확인적 요인분석 결과, 수렴타당도를 확인해 주는 개념신뢰도 (Construct Reliability, CR)는 인터넷게임장애 .94, 아버지와 자녀 간의 의사소통 방식 .71, 공격성 .87로 수용기준치 .7 이상을 만족하였다. 평균분산추출지수는 인터넷 게임장애는 .65, 아버지와 자녀 간의 의사소통 방식은 .57, 공격

Volume: 40, Article ID: e2018039
https://doi.org/10.4178/epih.e2018039

성 .63으로 수용기준 .5이상을 만족하여 수렴타당도가 있었다(Figure. 1).

Insert Figure 1 here

주요측정변수의 판별타당도는 표3의 대각선에 있는 평균분산추출이 대각선 하단에 있는 각 요인 간의 상관계수 제곱 값 보다 큰 값으로 판별타당도가 있었다(Table 3).

Insert Table 3 here

연구모형 적합도 검정(SEM)

공격성과 인터넷게임장애 위험성 간의 관계에서 아버지와의 의사소통 방식 매개효과 모형의 적합도 검정 결과 (x^2=201.52, df=87, x^2/df=2.32, GFI=.94, RMSEA=.06, CFI=.94, TLI=.92)로 매개효과 모델이 적합한 것으로 나타났다(Table 4).

Insert Table 4 here

직접효과, 간접효과, 총 효과

Volume: 40, Article ID: e2018039
https://doi.org/10.4178/epih.e2018039

구조방정식 모형 분석결과 공격성이 인터넷게임장애 위험성에 영향을 미치는 직접효과는 0.29으로 통계적으로 유의하였고($p<.001$), 공격성과 인터넷게임장애 위험성 간의 관계에서 아버지와 자녀 간의 의사소통 방식의 간접효과는 0.13이었다. 부트스트랩을 이용하여 간접효과의 유의성을 파악한 결과, 공격성과 인터넷게임장애 위험성에서 아버지와 자녀 간의 의사소통 방식이 매개하는 경로는 통계적으로 유의하였다($p<.001$). 따라서, 공격성이 인터넷게임장애 위험성에 미치는 영향을 아버지와 자녀 간의 의사소통 방식이 부분적으로 매개하는 것이 확인되었고, 총 효과는 0.42이었다 ($p<.001$) (Table 5).

Insert Table 5 here

DISCUSSION

본 연구에서 Buss와 Perry (1992)의 AQ 도구로 측정한 공격성 평균은 57.34(±15.00)로 다른 연구결과에서 보다는 다소 낮은 것으로 나타났다 [24]. AQ척도에서 공격성은 타인에게 상처나 상해를 입히려는 경향성으로 타인에게 신체적·언어적 공격행동 및 위협적인 사고와 정서를 포함한 것으로 정의하였다. 이는 청소년기에 나타나는 호르몬이나 전환기 환경 적응 등의 과정에서 생기는 충동성이나 공격적 욕구를 반영한다고 볼 수 있다. 본 연구 참여자는 중학교 1학년으로 아직 사춘기의 정점에 도달하지 않았을 것이라고 추정해 보았을 때 중학생 전체를 대상으로 수행한 연구나 고등학생을 대상으로 수행한 타 연구에서 보다는 공격성 점수가 낮을 것으로 여겨진다. 11-18세 청소년 5151명을 대상으로 수행한 코호트 연구결과, 신체적 공격성은 15세 전후, 사회적 공격성은 14세 전후에 최고조에 달한 것으로 나타났으며, 청소년기의 공격성 증가는 청소년 초기에서는 낮았다가 점점 증가하여 나이가 들면서 감소하는 추세를 보였다 [25].

공격성은 환경적 요인보다는 생물학적인 요인의 영향이 더 큰 것으로 보고되었다 [26]. 뇌영상 연구 결과, 공격성은 내측 전전두엽 피질과 외측 전두엽 피질의 회백질 부피 감소와 연관성이 있었다. 전전두엽 영역에서 회백질 감소는 정신병 진단과 상관없이 공격적인 행동과 연관성 있었고 이는 전두엽의 회백질 부피는 유전적인 요소가 많은 작용을 하고 있기 때문에 공격성이 생물학적 요인에 영향을 받고 있음을 시사하고 있다 [27]. 쌍둥이 장기추적 연구 결과에서도 사회적 공격성은 유전적 요인이 주요하게 작용하고 있다는 결과를 보여주었다 [28,29].

DSM-5에서는 9가지 중 5가지 이상 증상이 있을 경우, IGD로 진단되는 것으로 제안하고 있다. IGUESS는 점수범위는 0-27점으로 인터넷게임장애 위험의 절단점은 10점이상이다. IGUESS 에서는

Volume: 40, Article ID: e2018039
https://doi.org/10.4178/epih.e2018039

증상이 "자주" 인 경우 2점으로 평가되므로 10점이상의 의미는 DSM-5 IGD 진단기준 9개 중 5가지에 해당될 수 있다 [21]. 본 연구에서 절단점 10점 이상은 9.0%이었다.
청소년 시기의 공격성과 인터넷게임장애 위험성 사이에 유의한 연관성이 있었는데(r=.32, p<.001), 이러한 결과는 공격성이 인터넷게임장애 위험성에 영향을 미친다는 선행연구 결과와 일치하였다 [30-32]. 따라서, 청소년시기의 공격성이 높으면 인터넷게임장애 위험군에 포함될 가능성이 높음을 의미한다.
청소년의 공격성과 인터넷게임장애 위험성과의 관계에서 부모와의 의사소통방식이 위험성을 낮출 수 있는지를 확인한 본 연구에서 어머니와의 의사소통 방식은 청소년의 공격성과 인터넷게임장애 위험성과의 관계에서 매개효과가 없는 것으로 나타났고, 아버지와의 의사소통 방식만이 매개효과가 있었다.
구조방정식 분석결과 공격성이 인터넷게임장애 위험성에 미치는 총효과는 0.42였으며 이 중 직접효과는0.29, 아버지와 자녀의 의사소통 방식을 통해 매개하는 간접효과는 0.13였다. 표준화 경로계수의 0.1-0.3 일 경우는 적은 효과를 의미하고 0.3 이상일 경우 보통효과, 0.5 이상일 경우 큰 효과가 있는 것으로 판단하므로 청소년의 공격성과 인터넷게임장애 위험성과의 연관성은 보통 이상의 효과가 있는 것으로 해석할 수 있다. 아버지와 자녀간의 의사소통을 개방적으로 증진하는 것이 인터넷게임장애 위험성을 포함한 문제 행동에 기여할 수 있는지에 대한 전향적 무작위화 임상연구가 필요하다.
의사소통은 복잡하고 역동적인 상호작용 과정이다. 부모역할비교 이론에 따르면, 전통가족에서의 아버지는 가정의 대표자로서 '도구적인 역할'을 수행하는 반면, 어머니는 정서적 욕구를 충족시키는 '표현적인 역할'을 담당한다. 양육과정에서 아버지는 자녀의 행동을 통제하는 통제적 역할을 하며, 어머니는 말이나 행동, 신체접촉을 통해 온화함을 제공하는 양육적 역할을 담당한다. 발로 차거나 무는 등의 신체적인 폭력이 사회적으로 받아들여지지 않는다는 것을 아버지와의 놀이를 통해 배우며, 소심함과 공격성 사이에 균형을 배울 수 있다[33]. 부모의 성별에 따라 자녀에게 다른 영향을 제공한다는 점에서 인터넷게임장애 위험성의 경우 아버지와 더 연관성이 있을 가능성이 있다. 영국의 출생코호트 추적 조사 결과 청소년 초기에 아버지의 관여와 태도는 자녀의 정신건강에 중요한 영향을 미쳤다 [34]. 일반적으로 아이들에게 어머니의 영향이 큰 것으로 알려져 있으나, 공격성과 인터넷게임장애 위험성과의 연관성에서 부친의 영향이 컸던 결과는 연구대상자가 중학교 1학년으로 구성되었기 때문일 수도 있다. 본 연구 결과가 특정 시기에 나타나는 현상인지 청소년기 전반에 나타나는지에 대해서는 향후 연구가 필요하다.
환경적 요인에 따른 공격성의 영향은 성별에 따라 차이가 있는 것으로 알려져 있으며 여성의 경우 환경의 영향을 덜 받는 것으로 보고되었다 [29]. 공격성과 인터넷게임중독 연관성에서 아버지와 자녀간의 의사소통 매개효과가 남녀간에 차이가 있는지를 확인하기 위하여 성별로 층화하여 다집단 구조방정식 모형을 분석하였으나 남녀간의 차이가 없이 남녀 모두에서 아버지와 자녀간의 의사소통방식이 매개요인으로 작용하였다 (결과는 제시하지 않음).

본 연구의 제한점으로 첫째, 단면조사연구이기 때문에 청소년의 공격성, 부모-자녀간의 의사소통 방식 및 인터넷게임장애 간의 시간적 선후관계를 볼 수 없다는 점이다. 인과관계 규명을 위해 추후 종단연구를 통해 증명하는 것을 제언한다.
둘째, 인터넷게임장애의 진단기준이 아직 명확하게 규명되지는 않아 오분류의 가능성이 있지만,

본 연구는 DSM-5에서 추가 연구 필요 상태(Conditions for Further Study)로 분류된 인터넷게임장애 기준을 적용한 IGUESS로 측정하여 신뢰도와 타당도를 높이고자 하였다. IGUESS가 자가보고에 의해 평가되었기 때문에 위양성과 위음성의 가능성을 배제할 수 없다.
셋째, 본 연구결과는 서울 일부 지역의 4개 중학교 대상 편의 표집으로 진행되었고 특히 중학교 1학년으로 구성되었기 때문에 전체 청소년 대상으로 일반화하기 어려운 한계점이 있다.
본 연구의 장점은 부모와 자녀와의 의사소통에서 부모를 구분하지 않고 의사소통 효과를 분석한 연구와 달리 본 연구에서는 아버지와 어머니를 구분하여 자녀와의 의사소통 매개효과를 구조방정식을 이용하여 평가한 점이다.
본 연구에서는 청소년 시기의 공격성은 인터넷게임장애 위험성과 연관성이 있으며 청소년들과 아버지와의 의사소통 방식이 매개효과가 있으므로, 아버지와 자녀 간의 개방적이고 긍정적인 의사소통을 하기 위한 노력이 필요하다는 점을 시사하고 있다.

ACKNOWLEDGEMENTS

This study was supported by a grant from the Korean Mental Health Technology R&D Project, Ministry of Health and Welfare, Republic of Korea (HM14C2603).

CONFLICT OF INTEREST

The authors have no conflicts of interest to declare for this study.

REFERENCES

[1] Duke SA, Balzer BW, Steinbeck KS. Testosterone and its effects on human male adolescent mood and behavior: a systematic review. J Adolesc Health 2014;55:315-22. DOI: 10.1016/j.jadohealth.2014.05.007.
[2] Orpinas P FR. The Aggression Scale. The Journal of Early Adolescence. 2001.
[3] Liu J, Lewis G, EvansL. Understanding Aggressive Behavior Across the Life Span. J Psychiatr Ment Health Nurs 2013;20:156-68.
[4] Granic I, Dishion TJ, Hollenstein T. The family ecology of adolescence: A dynamic systems perspective on normative development. Blackwell handbook of adolescence 2003:60-91.
[5] Cheung C. S. S. PEM. Why does parents' involvement enhance children's achievement? The role of parent-oriented motivation. . J Educ Psychol 2012;104:820.
[6] Cava MJ, Buelga S, Musitu G. Parental communication and life satisfaction in adolescence. Span J Psychol 2014;17:E98. DOI: 10.1017/sjp.2014.107.
[7] Collins WE, Newman BM, McKenry PC. Intrapsychic and interpersonal factors related to adolescent psychological well-being in stepmother and stepfather families. Journal of

Family Psychology 1995;9:433.

[8] Luk JW, Farhat T, Iannotti RJ, et al. Parent-child communication and substance use among adolescents: do father and mother communication play a different role for sons and daughters? Addict Behav 2010;35:426-31. DOI: 10.1016/j.addbeh.2009.12.009.

[9] Khaokhajorn P, Samipak S, Nithithanasilp S, et al. Production and secretion of naphthoquinones is mediated by the MFS transporter MFS1 in the entomopathogenic fungus Ophiocordyceps sp. BCC1869. World Journal of Microbiology and Biotechnology 2015;31:1543-54.

[10] Brooks F, Magnusson J, Klemera E, et al. HBSC England national report: Findings from the 2014 HBSC study for England. Hatfield: University of Hertfordshire 2015.

[11] Lin F, Zhou Y, Du Y, et al. Abnormal white matter integrity in adolescents with internet addiction disorder: a tract-based spatial statistics study. PloS one 2012;7:e30253.

[12] Arain M, Haque M, Johal L, et al. Maturation of the adolescent brain. Neuropsychiatr Dis Treat 2013;9:449-61. DOI: 10.2147/ndt.s39776.

[13] KIS A. Survey on the Internet usage. In: Ministry of Science IaFP, editor 2016.

[14] Gunuc S. Peer Influence in Internet and Digital Game Addicted Adolescents: Is Internet/Digital Game Addiction Contagious? International Journal of High Risk Behaviors and Addiction 2016.

[15] Kuss DJ. Internet gaming addiction: current perspectives. Psychology research and behavior management 2013;6:125.

[16] Kuss DJ, Lopez-Fernandez O. Internet addiction and problematic Internet use: A systematic review of clinical research. World journal of psychiatry 2016;6:143.

[17] Jeong H, Yim HW, Jo SJ, et al. Study protocol of the internet user Cohort for Unbiased Recognition of gaming disorder in Early adolescence (iCURE), Korea, 2015-2019. BMJ Open 2017;7:e018350. DOI: 10.1136/bmjopen-2017-018350.

[18] Wolf EJ, Harrington KM, Clark SL, et al. Sample Size Requirements for Structural Equation Models: An Evaluation of Power, Bias, and Solution Propriety. Educ Psychol Meas 2013;76:913-34. DOI: 10.1177/0013164413495237.

[19] Buss AH, Perry M. The aggression questionnaire. Journal of personality and social psychology 1992;63:452.

[20] Seo S, Kwon S. Validation study of the Korean version of the aggression questionnaire. Korean J Clin Psychol 2002;21:487-501.

[21] Jo SJ, Yim HW, Lee HK, et al. The Internet Game Use-Elicited Symptom Screen proved to be a valid tool for adolescents aged 10–19 years. Acta Paediatrica 2018;107:511-6.

[22] Barnes H, Olson DH. Parent-adolescent communication, family inventories. Family social science, University of Minnesota 1982.

[23] Min H. Circumplex model and parent-child communication [master's thesis]. Seoul: Yonsei University 1990:1-70.

[24] Moon JY, Kim SW, Lee KE, et al. Correlation between Aggression and Health Behaviors of

Korean High School Students. Korean Journal of Clinical Pharmacy 2014;24.

[25] Karriker-Jaffe KJ, Foshee VA, Ennett ST, et al. The development of aggression during adolescence: Sex differences in trajectories of physical and social aggression among youth in rural areas. Journal of abnormal child psychology 2008;36:1227.

[26] Niv S, Tuvblad C, Raine A, et al. Aggression and Rule-breaking: Heritability and stability of antisocial behavior problems in childhood and adolescence. J Crim Justice 2013;41. DOI: 10.1016/j.jcrimjus.2013.06.014.

[27] Coccaro EF, Cremers H, Fanning J, et al. Reduced frontal grey matter, life history of aggression, and underlying genetic influence. Psychiatry Res Neuroimaging 2018;271:126-34. DOI: 10.1016/j.pscychresns.2017.11.007.

[28] Porsch RM, Middeldorp CM, Cherny SS, et al. Longitudinal heritability of childhood aggression. Am J Med Genet B Neuropsychiatr Genet 2016;171:697-707. DOI: 10.1002/ajmg.b.32420.

[29] Slawinski BL, Klump KL, Burt SA. The etiology of social aggression: a nuclear twin family study. Psychol Med 2018:1-8. DOI: 10.1017/s0033291718000697.

[30] Grüsser SM, Thalemann R, Griffiths MD. Excessive computer game playing: evidence for addiction and aggression? CyberPsychology & Behavior 2006;10:290-2.

[31] Lim J. The Mediation Model Verification of Private Self-consciousness on Game Addiction and Aggression. The Journal of the Korea Contents Association 2016;16:250-61.

[32] Lee J, Choi KS. Longitudinal Structural Equation Modeling of Internet Game and Aggression in Children. Open Journal of Nursing 2015;5:426.

[33] Yogman M, Garfield CF. Fathers' Roles in the Care and Development of Their Children: The Role of Pediatricians. Pediatrics. 2016;138(1). pii: e20161128. doi: 10.1542/peds.2016-1128. Epub 2016 Jun 13.

[34] Opondo C, Redshaw M, Quigley MA. Association between father involvement and attitudes in early child-rearing and depressive symptoms in the pre-adolescent period in a UK birth cohort. J Affect Disord 2017;221:115-22. DOI: 10.1016/j.jad.2017.06.010.
